# Active Surveillance for Medically Inoperable Stage IA Lung Cancer in the Elderly

**DOI:** 10.7759/cureus.3472

**Published:** 2018-10-22

**Authors:** Hyunsoo J No, Nataniel H Lester-Coll, David J Seward, Nikoletta Sidiropoulos, Havaleh M Gagne, Carl J Nelson, Garth W Garrison, C. Matthew Kinsey, Steven H Lin, Christopher J Anker

**Affiliations:** 1 Radiation Oncology, Larner College of Medicine at the University of Vermont, Burlington, USA; 2 Radiation Oncology, University of Vermont Cancer Center, Burlington, USA; 3 Pathology and Laboratory Medicine, Larner College of Medicine at the University of Vermont and the University of Vermont Health Network, Burlington, USA; 4 Pulmonology and Critical Care Medicine, University of Vermont Medical Center, Burlington, USA; 5 Pulmonary and Critical Care Medicine, University of Vermont Medical Center, Burlington, USA; 6 Radiation Oncology, The University of Texas MD Anderson Cancer Center, Houston, USA

**Keywords:** non-small cell lung cancer, elderly, active surveillance

## Abstract

Objectives

Treatment for stage IA lung cancer may be too aggressive an approach in elderly patients with competing co-morbidities. We report outcomes for those electing active surveillance (AS) and investigate factors that may predict indolent disease.

Materials and methods

Retrospective review was performed for 12 consecutive patients, ≥70 years old, with medically inoperable stage IA, T1N0M0 lung cancer and significant co-morbidities, who chose AS with radiation therapy (RT) reserved for clear disease progression. Collected data included Charlson-Deyo Comorbidity Index (CDCI) grades, histology, and tumor size changes. Volume doubling time (VDT) calculations used a modified Schwartz equation.

Results

Fifteen nodules underwent AS in 12 patients; three patients had more than one nodule. Median age of all patients was 78 (range, 71–85). All patients’ CDCI grades were ≥1, 7 were ≥2. Eleven of 12 patients were deemed to be at high-risk for falls. Twelve nodules in 12 patients were biopsied; adenocarcinoma the prevailing common (47%) histology. The median, one, two and three year patient freedom-from-RT values were 21.4 months (95% CI: 11.6-not reached), 81%, 43%, and 29%, respectively. Median VDT of treated vs. untreated nodules was 189 days (range, 62-infinite) vs. 1153 days (range, 504-infinite), respectively. No patient progressed regionally or distantly, and there have been no cancer-related deaths. Due to cardiovascular events, two patients died and one remains on hospice. Median duration of AS for those still continuing computed tomography (CT) surveillance is 35.1 months.

Conclusion

Selected elderly patients with stage IA lung cancer and significant co-morbidities may undergo AS without detriment in outcome. Prospective AS studies are warranted.

## Introduction

Untreated stage I lung cancer generally portends a poor prognosis, with median overall survival (OS) of 9–14 months [1]. However in elderly populations, particularly those with significant comorbidities, treatment decisions must weigh a cancer’s aggressiveness with competing risks of death. There are an increasing number of retrospective studies noting the safety and efficacy of stereotactic ablative radiotherapy (SABR) for early-stage non-small cell lung cancer (NSCLC) in the elderly [2-7]. However, radiation therapy (RT)-associated morbidity could negatively impact quality-of-life (QOL), and, although rare, SABR has been implicated in serious complications [8-10], which can be more impactful for those with waning baseline QOL.

Lung cancer screening using low-dose computed tomography (CT) scans was associated with a 20% reduction in mortality in the National Lung Screening Trial Research Team (NLST) trial [11]. Although less than 9% of the patient population in the NLST trial was >70 years old, the US Preventive Services Task Force advocates lung cancer screening and treatment for patients up to 80 years old [12]. With increased screening, it can be expected that an increasing number of elderly patients with significant comorbidities will be found to have early-stage NSCLC; however, survival benefit decreases with increasing age. Of note, over 95% of patients in the NLST had negative biopsies, putting many patients without cancer at risk for procedural complications [11]. Additionally, there has been an associated overtreatment rate of ~18% [13]. Overtreatment may be even more prevalent in elderly populations, where larger proportions of asymptomatic nodules are found incidentally [14].

Prognostic factors, i.e., tumor volume doubling time (VDT), positron emission tomography [15] maximum standardized uptake value (PET SUV), and solid component size [16], may improve predictions regarding who would be best managed with active surveillance (AS): AS is defined as following patients closely with intent to provide definitive treatment, if necessary. This approach would allow frailer, older individuals to avoid, or delay, treatment-associated toxicity without compromising tumor treatability or cancer outcomes. Here, we report the first series to our knowledge investigating AS for elderly patients with incidentally discovered medically inoperable stage IA lung cancer.

## Materials and methods

Twelve consecutive patients were retrospectively reviewed with medically inoperable pathologically confirmed stage IA, T1N0M0 lung cancer [17], who chose AS between 2014 and 2017, with treatment reserved for clear radiographic progression of disease. Follow-up chest CTs were recommended as frequently as every two months, and up to every six months if disease was approximately stable. Clinical data included Karnofsky Performance Score (KPS), Charlson-Deyo Comorbidity-Index (CDCI), fall-risk status, tumor size, PET SUV Max, and pathologic information. Freedom-from-RT was evaluated by the Kaplan-Meier method, with duration of AS follow-up recorded from radiographic nodule detection. Post-treatment follow-up was recorded from last radiation treatment date to most recent follow-up CT. VDT calculations used a modified Schwartz equation for exponential growth. All procedures followed were in accordance with the ethical standards of the responsible committee on human experimentation (institutional and national) and with the Helsinki Declaration of 1975, as revised in 2008. Informed consent was obtained from all patients for being included in the study.

## Results

This study included 12 inoperable patients with 15 total lung nodules (Table 1). Median age at diagnosis was 78 (range, 71–85), and median initial KPS was 70. Eleven of 12 patients were deemed to be at high risk for falling and were advised on preventative measures. All nodules were asymptomatic and found incidentally. Biopsy of lesions was performed by both fine needle aspiration and core needle biopsy for all patients, and all patients were staged by PET scan. Endobronchial ultrasound (EBUS) staging was performed for patients with hilar or mediastinal nodes that were enlarged or exhibited high SUV max values on PET scan. Fourteen lesions in 10 patients were staged T1a [17], the remaining two (lesions 6 & 7) were T1b. All nodules were 90–100% solid. Tumors with a VDT of ≥400 days had a median numerically lower SUV if no biopsy was performed before PET-imaging.

**Table 1:** Non-operable stage IA lung cancer patients under active surveillance (AS). AS: Active surveillance; Pt: Patient; Dx: Diagnosis; RUL: Right-upper lobe; RML: Right-middle lobe; RLL: Right-lower lobe; LUL: Left-upper lobe; LLL: Left-lower lobe; Adeno: Adenocarcinoma; SCC: Squamous-cell carcinoma; NEC: Neuroendocrine carcinoma; NSCLC: Non-small cell lung cancer; Non-Bx: Not biopsied; Non-Dx: Biopsy non-diagnostic; OBV: Observation; SABR: Treated with SABR; 3DCRT: Three-dimensional conformal radiation therapy; AS follow-up: Active surveillance follow-up measured from time of nodule detection; ∞: No lesion size change in subsequent CTs; StdDev: Standard deviation; VDT: Volume doubling time. *Patients listed in order of decreasing VDT. **18.9 months median for all patients; 35.1 months median for living patients continuing AS; 21.4 months median of AS time for patients receiving treatment.

Pt*	Sex	Age at dx	CDCI	KPS initial	KPS recent	Lesion	Site	Pathology	Max initial size [mm]	Max final size [mm]	VDT [Days]	Initial PET SUV Max	#Days PET obtained post biopsy	Management status	AS Follow- up [Months]	Follow-up post-treatment [Months]
1	M	73	2	60	0	A	LLL	NEC	16.0	15.9	∞	1.73	N/A	OBV	11.3	-
2	M	78	2	80	60	A	LLL	Adeno	15.0	15.0	∞	1.80	24	OBV	12.0	-
3	F	76	2	80	80	A	RML	NSCLC	19.8	20.4	∞	5.90	N/A	3DCRT	6.4	9.8
4	F	77	2	90	90	A	LUL	NEC	17.7	17.9	3380	6.20	N/A	OBV	19.6	-
5	F	81	2	70	60	A	RLL	Adeno	8.0	12.6	1320	3.30	24	OBV	42.3	-
6	M	74	1	60	50	A	RLL	Adeno	14.4	19.5	532	1.70	33	OBV	28.5	-
7	M	84	2	60	0	A	RUL	NSCLC	26.0	30.0	504	12.10	26	OBV	5.0	-
8	M	82	1	70	70	A	LLL	Adeno	13.7	18.5	317	8.20	N/A	3DCRT	11.6	4.2
9	F	83	1	70	60	A	RUL	Adeno	12.0	22.2	242	1.90	N/A	SABR	18.1	16.5
10	F	71	2	70	70	A	RUL apex	Adeno	12.0	28.0	229	4.44	7	SABR	25.2	22.8
						B	RUL posterior	Non-Bx	13.0	16.6	2700	2.42	N/A	OBV	41.6	-
11	M	85	2	90	70	A	RUL	SCC	25.0	39.0	148	5.60	3	3DCRT	20.7	16.0
						B	RLL	Non-Bx	8.0	18.0	97	2.37	N/A	SABR	12.5	20.7
12	M	75	1	90	60	A	RML	Adeno	8.0	16.0	985	1.07	11	OBV	131.4	-
						B	LUL	Non-Bx	3.0	12.0	62	0.97	N/A	SABR	21.4	45.4
Median		78	2	70	60	N/A	N/A	N/A	13.7	18.4	504	3.15	24	N/A	18.9**	16.5
Mean		78	N/A	N/A	N/A	N/A	N/A	N/A	14.6	21.7	540	4.19	18	N/A	27.6	19.3
Range		71 – 85	1-2	60-90	0-90	N/A	N/A	N/A	3.0-26.0	12.0-40.6	62-∞	1.07-12.1	3-33	N/A	5.0-131 .4	4.2-45.4
Std Dev		4.7	N/A	N/A	N/A	N/A	N/A	N/A	6.5	8.9	488	2.77	10.4	N/A	N/A	N/A

**Figure 1:**
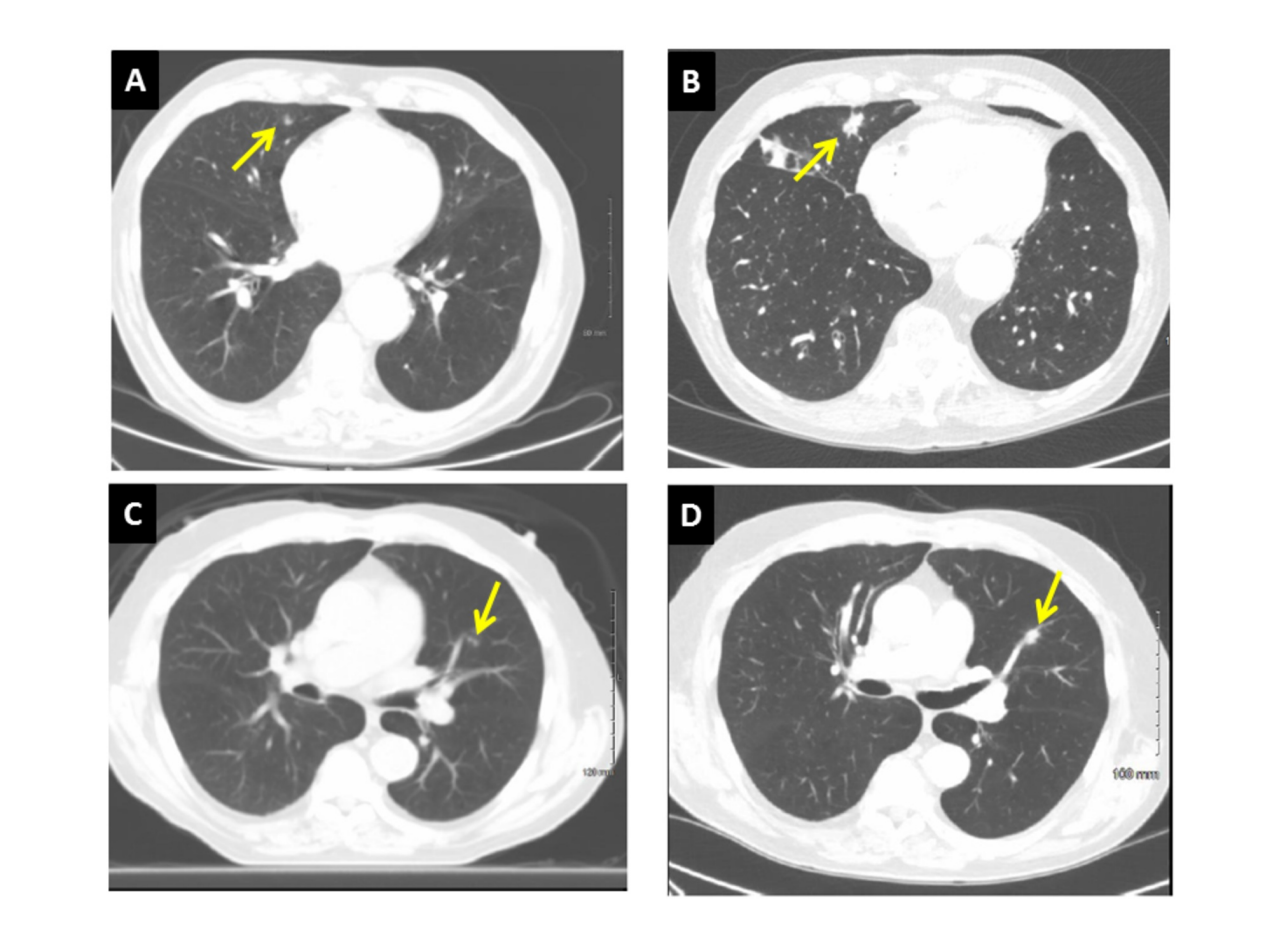
Patient 12. (A) RML adenocarcinoma, initially 8 x 5 mm. (B) 11.0 years later, measuring 16 x 15 mm (VDT = 985 days); AS continues. (C) Unbiopsied LUL nodule, initially 3 x 3 mm. (D) 21.3 months later, measuring 12 x 9 mm (VDT = 62 days); SABR delivered. RML: Right-middle lobe; VDT: Volume doubling time; AS: Active surveillance; LUL: Left-upper lobe; SABR: Stereotactic ablative radiotherapy.

**Figure 2:**
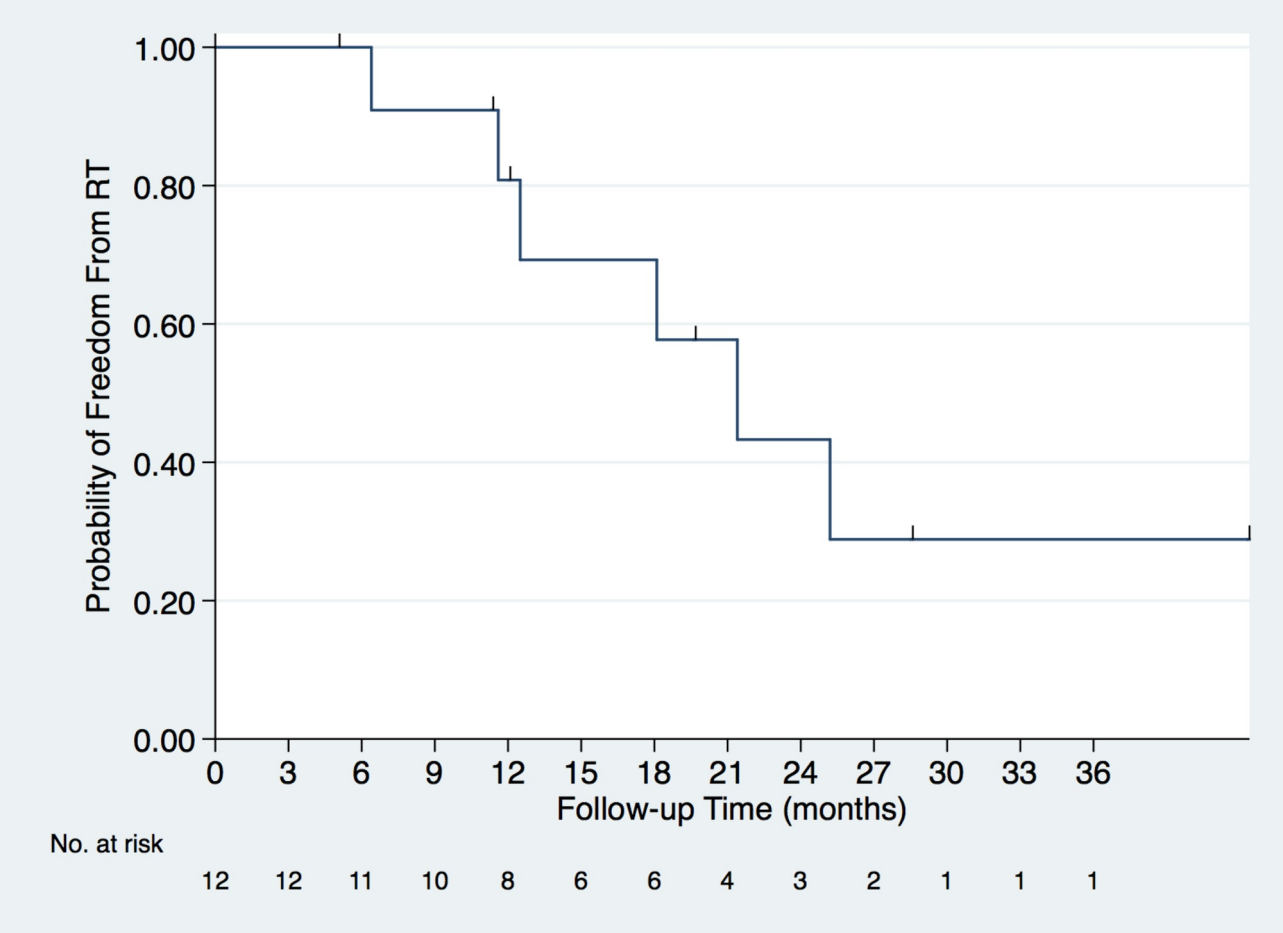
Kaplan-Meier estimated survival curve showing freedom-from-RT among the 12 patients. Seven nodules in six patients received RT. Median, one, two and three year patient freedom-from-RT values were 21.4 months (95% CI: 11.6-not reached), 81%, 43%, and 29%, respectively. RT: Radiation therapy

Seven tumors in six patients were treated due to tumor progression; three patients had single nodules and the remaining three patients had two. Median duration of AS for those still continuing CT-surveillance is 35.1 months, range (12–131.4), excluding those deceased (n = 2), on hospice (n = 1), or treated with RT (n = 7). The reason for the long follow-up time for patient 12 is that he initially declined therapy following diagnosis, but then chose AS after the option started being offered in 2014. Follow-up chest CTs were performed at a median interval of four months (range, 2–6). Two of seven treated lesions were not biopsy-proven, but were growing more rapidly than biopsy-proven cancers of the same patient (Figure 1A, 1D). The median, one, two and three year patient freedom-from-RT values were 21.4 months (95% CI: 11.6-not reached), 81%, 43%, and 29%, respectively (Figure 2). Indications for treatment were based on concern from large increases in tumor diameter, given the lack of existing data on a VDT-directed AS approach. Median VDT of nodules treated because of observed tumor growth was 189 days (range, 62–infinite) vs. 1153 days (range, 504–infinite) for untreated nodules. The only patient to request RT before it was encouraged was patient 3, who reported family concerns led to the decision, despite the patient’s comfort with AS and an infinite VDT (no growth observed in 6.4 months). SABR involved 50 Gy in five fractions delivered every other day. Although eligible by size criteria for SABR, due to concerns for potential cardiac and chest wall toxicity from the more aggressive SABR fractionation, patients 3, 8, and 11 opted for 15-fraction courses of daily radiation (52–52.5 Gy). Toxicities included grade 1 dermatitis (n = 1) and grade 1 (n = 5) fatigue.

No patient progressed regionally or distantly, and there have been no cancer-related deaths. Due to debilitating cardiovascular disease, patient 2 died during AS (15 months post-nodule detection). Patients 3 and 7 transitioned to hospice during AS; patient 7 has since passed away from congestive heart failure (19 months post-nodule detection).

## Discussion

In “Being Mortal,” Gawande highlights a patient with a recently discovered pulmonary nodule. After feeling concern regarding a geriatrician’s dismissal of the pulmonary nodule, Gawande learned “...the single most serious threat she faced was not the lung nodule… it was falling [18].” Similarly, all patients in this series had incidentally discovered lung tumors amidst advanced age and competing comorbidities of larger concern. Coincidently, 92% were deemed high-risk for falls. The primary goals of care for this patient population focused on avoiding any side effects that could result in any loss of independence, and noting the asymptomatic character of their malignancies, AS was elected by the individuals of this group. In the management for early-stage lung cancer in medically inoperable elderly patients, SABR is an oft-elected modality, as it is associated with increased OS compared to no treatment [1], but those receiving SABR have an expected median OS of just 17.7–29 months, compared to 10 months via observation [1,2]. SABR is associated with three-year actuarial regional and distant failure of 8–10% and 9–23%, respectively. Three-year OS and cause-specific survival (CSS) rates are reported at 42–57.5% and 50.4–90%, respectively, indicating many patients die for reasons other than cancer, and, even with treatment, cancer-related fatalities may occur [2].

Scholten et al. showed CT-monitoring is suitable for persistent sub-solid or partially solid nodules, as they rarely develop into clinically manifesting malignancies unexpectedly [16]. Our data suggests that preservation of curative potential is possible in an AS program, even for entirely solid nodules. All 15 nodules consisted of 90–100% solid components, with no regional/distant progression at a median AS time of 35.1 months for all living patients. Therefore, we did not find compromise of oncologic outcomes from AS. Survival of patients in this series is comparable to treated patients of other SABR series [1,2].

Although SABR has been established as safe and effective for early stage NSCLC, there are still potential side-effects. Kreinbrink et al. did not report any chest wall toxicity, but most tumors were small, dosimetry was favorable, and median follow-up was shorter than median time-to-chest wall toxicity reported by Thibault et al. [2,10]. In other series, the incidence of grade 1 and 2 chest wall toxicity has been reported at 25% and 4%, respectively. For osteoporotic elderly patients, Thibault et al. noted a two-year fracture rate >60%, with events continuing to occur beyond two years [10]. Following SABR, rates of grade 2 and ≥3 pneumonitis have been reported in up to 20% and 10% of patients, respectively, with even grade 2 toxicities affecting abilities to perform basic activities of daily living (ADL) [2].

Limitations of this study include its retrospective nature and limited sample size. Not all lesions underwent biopsy, with diagnosis based on PET-avidity and growth, but all patients had at least one pathologically proven lung cancer. Treatment indications were based on a fast diameter-based measurements growth rate and not VDT, given the lack of data to guide a VDT-directed approach.

Despite the noted limitations, this series is valuable as the first report of a potentially effective treatment algorithm, minimizing toxicity while maintaining the benefit of treatment at an early stage. Our data suggests that AS may be a reasonable option for selected stage I NSCLC medically inoperable patients, without compromising outcomes. As SABR is shown to provide a median OS of 17.7–29 months in medically inoperable elderly patients [1], comparatively, this series has provided a median duration follow-up of 35.1 months for those who elected AS and continuing CT-surveillance. Remarkably, patient 12 has been followed for over 11 years without requiring treatment to the biopsy-proven malignancy in his right-middle lobe (RML), and has had no regional or distant spread of disease. Treatment-related toxicities are reported to be low in the elderly including those up to 90 years old [2]; however, there is still a risk associated with putting people into treatment, and the pendulum may be swinging towards overtreatment. Despite the low risk, risk of toxicity still exists, which can be ever more significant for those with senescent independence.

The importance of AS appears to be increasing beyond prostate to include breast and thyroid [19], and we believe this process deserves further evaluation for frail, elderly patients with lung cancer. Our data has formed the basis for a currently open prospective study, where patients are offered SABR versus continued AS based on tumor VDT and size, incorporating a concurrent analysis of biomarkers, quality of life measures, and allowing characterization of outcomes with a larger study population.

## Conclusions

Untreated stage I lung cancer generally portends a poor prognosis, however in elderly populations, particularly those with significant comorbidities, treatment decisions must weigh a cancer’s aggressiveness with competing risks of death. Selected elderly patients with stage IA lung cancer may benefit from an active surveillance approach. This study included 12 inoperable patients with 15 total lung nodules who underwent AS. Median duration of AS for those still continuing CT-surveillance is 35.1 months. Of significance, no patient progressed regionally or distantly, and there have been no cancer-related deaths.

This series is valuable as the first report of a potentially effective treatment algorithm, minimizing toxicity while maintaining the benefit of treatment at an early stage. Our data suggests that AS may be a reasonable option for selected stage I NSCLC medically inoperable patients, without compromising outcomes.

## References

[1] Nanda RH, Liu Y, Gillespie TW (2015). Stereotactic body radiation therapy versus no treatment for early stage non-small cell lung cancer in medically inoperable elderly patients: a National Cancer Data Base analysis. Cancer.

[2] Kreinbrink P, Blumenfeld P, Tolekidis G, Sen N, Sher D, Marwaha G (2017). Lung stereotactic body radiation therapy (SBRT) for early-stage non-small cell lung cancer in the very elderly (≥80 years old): extremely safe and effective. J Geriatr Oncol.

[3] Haasbeek CJ, Lagerwaard FJ, Antonisse ME, Slotman BJ, Senan S (2010). Stage I nonsmall cell lung cancer in patients aged ≥75 years. Cancer.

[4] Sandhu AP, Lau SK, Rahn D (2014). Stereotactic body radiation therapy in octogenarians with stage I lung cancer. Clin Lung Cancer.

[5] Hayashi S, Tanaka H, Kajiura Y, Ohno Y, Hoshi H (2014). Stereotactic body radiotherapy for very elderly patients (age, greater than or equal to 85 years) with stage I non-small cell lung cancer. Radiat Oncol.

[6] Brooks ED, Sun B, Zhao L (2017). Stereotactic ablative radiation therapy is highly safe and effective for elderly patients with early-stage non-small cell lung cancer. Int J Radiat Oncol Biol Phys.

[7] Giuliani M, Hope A, Guckenberger M (2017). Stereotactic body radiation therapy in octo- and nonagenarians for the treatment of early-stage lung cancer. Int J Radiat Oncol Biol Phys.

[8] Bahig H, Filion E, Vu T (2016). Severe radiation pneumonitis after lung stereotactic ablative radiation therapy in patients with interstitial lung disease. Pract Radiat Oncol.

[9] Haseltine JM, Rimner A, Gelblum DY (2016). Fatal complications after stereotactic body radiation therapy for central lung tumors abutting the proximal bronchial tree. Pract Radiat Oncol.

[10] Thibault I, Chiang A, Erler D (2016). Predictors of chest wall toxicity after lung stereotactic ablative radiotherapy. Clin Oncol (R Coll Radiol).

[11] Aberle DR, Adams AM, Berg CD (2011). Reduced lung-cancer mortality with low-dose computed tomographic screening. N Engl J Med.

[12] Humphrey LL, Deffebach M, Pappas M (2013). Screening for lung cancer with low-dose computed tomography: a systematic review to update the US preventive services task force recommendation. Ann Intern Med.

[13] Patz EF Jr, Pinsky P, Gatsonis C (2014). Overdiagnosis in low-dose computed tomography screening for lung cancer. JAMA Intern Med.

[14] Gould MK, Tang T, Liu IL (2015). Recent trends in the identification of incidental pulmonary nodules. Am J Respir Crit Care Med.

[15] Hofheinz F, Langner J, Petr J (2012). A method for model-free partial volume correction in oncological PET. EJNMMI Res.

[16] Scholten ET, de Jong PA, de Hoop B (2015). Towards a close computed tomography monitoring approach for screen detected subsolid pulmonary nodules?. Eur Respir J.

[17] Edge S, Byrd DR, Compton CC, Fritz AG, Greene F, Trotti A (2010). AJCC Cancer Staging Handbook: From the AJCC Cancer Staging Manual. https://www.springer.com/in/book/9780387884424.

[18] Gawande A (2014). Being Mortal: Medicine and What Matters in the End.

[19] Haymart MR, Miller DC, Hawley ST (2017). Active surveillance for low-risk cancers - a viable solution to overtreatment?. N Engl J Med.

